# Prevalence of asymptomatic *Leishmania* infection and associated risk factors, after an outbreak in the south-western Madrid region, Spain, 2015

**DOI:** 10.2807/1560-7917.ES.2019.24.22.1800379

**Published:** 2019-05-30

**Authors:** Ana Victoria Ibarra-Meneses, Eugenia Carrillo, Javier Nieto, Carmen Sánchez, Sheila Ortega, Alicia Estirado, Pello Latasa Zamalloa, Juan Carlos Sanz, Luis García-Comas, María Ordobás, Javier Moreno

**Affiliations:** 1 WHO Collaborating Centre for Leishmaniasis, National Center for Microbiology, Instituto de Salud Carlos III, Majadahonda, Madrid, Spain; 2 Department of Epidemiology, Consejería de Sanidad de la Comunidad de Madrid, Madrid, Spain; 3 Regional Public Health Laboratory, Health Department of the Community of Madrid, Madrid, Spain

**Keywords:** asymptomatic infection, whole blood stimulated assay, interleukin-2, risk factors, Leishmania, epidemiological survey, epidemiology, leishmaniasis, outbreaks, Spain, surveillance

## Abstract

**Background:**

A large outbreak of leishmaniasis with 758 cutaneous and visceral leishmaniasis cases occurred in 2009 in Fuenlabrada, in the south-west of the Madrid region of Spain.

**Aim:**

We aimed to determine the prevalence of asymptomatic *Leishmania* infection after this outbreak, and its associated risk factors.

**Methods:**

A cross-sectional study of 804 healthy individuals living in Fuenlabrada who had no history of leishmaniasis, was conducted between January and July 2015. Asymptomatic infections were sought by either a combination of PCR, immunofluorescent antibody titre, and direct agglutination tests, or by whole blood stimulation assay (WBA) with interleukin-2 (IL-2) quantification.

**Results:**

Using the first approach, prevalence of asymptomatic individuals was 1.1% (9/804), while the second returned a value of 20.7% (143/804). Older age, being male, proximity to the park where the focus of infection was identified, and living in a detached house, were all strongly associated with the prevalence of asymptomatic infection.

**Conclusions:**

The true number of infected individuals may be underestimated if only serological methods are used. The combination of WBA with IL-2 quantification may allow to better determine the prevalence of asymptomatic *Leishmania* infection, which would be useful in establishing control measures and in quantifying their impact. In our study, the use of WBA with IL-2 quantification also helped establish the risk factors that influence exposure to and infection by *Leishmania*.

## Introduction

Leishmaniasis is one of the most neglected tropical diseases, yet it is associated with high mortality and morbidity [1]. Some 350 million people are at risk of becoming infected worldwide, while disease incidence is estimated at 2 million new cases every year [2]. Visceral leishmaniasis (VL; also known as kala-azar), is the most serious form of the disease, and is fatal if untreated. Mainly caused by *Leishmania donovani* and *L. infantum*, it is transmitted by the bite of infected female sand flies [1,2]. VL is distributed worldwide and is endemic in 80 countries across Asia, East Africa, South America and the Mediterranean region, including Spain.

In endemic areas, most people infected with *Leishmania* show no signs or symptoms of the disease. A number of studies have been performed to determine the number of asymptomatic individuals and of active cases of VL in different *L. donovani-* and *L. infantum-*endemic areas [3]. In Africa, the reported ratios of asymptomatic individuals and cases of active disease range from 2.4:1 (Sudan) to 5.6:1 (Ethiopia). In Asia, the ratio varies between 4:1 for Bangladesh and 8.9:1 for India and Nepal [4-6]. In South America, a ratio of 18:1 has been reported for Brazil. In Spain, a 50:1 ratio has been estimated [3]. This variation reflects the differences in parasite virulence and host characteristics, but perhaps also those associated with study design and the tests used to identify asymptomatic infection. Currently, the epidemiological role of the asymptomatic population is unknown. Mathematical modelling has suggested that transmission is maintained by asymptomatically infected hosts and that such parasite reservoirs could give rise to future epidemics [7]. It is thus essential to know the factors associated with asymptomatic infection if VL prevention and control strategies are to be properly designed and implemented [8].

The tests used to identify asymptomatic infection are mainly serological and include the direct agglutination test (DAT), the rk39 immunochromatographic test (rK39-ICT), and testing of immunofluorescent antibody titres (IFAT); all these detect *Leishmania*-specific antibodies [4]. Parasitological diagnosis, however, remains the gold standard in the diagnosis of leishmaniasis because of its high specificity. Molecular methods such as PCR have also been used to identify asymptomatic infection but less frequently, due to the difficulty in performing such analyses in the field. Cellular assays, such as the leishmanin skin test (LST) have also been widely used in epidemiological surveys [9-14]. The whole blood stimulation assay (WBA), which involves the use of soluble *Leishmania* antigen (SLA) and the subsequent detection of cytokines and chemokines in stimulated plasma, has recently been proven useful for identifying asymptomatic infection by *L. infantum* and *L. donovani* [15-19]. Its simplicity, speed and the lack of any need for sterile conditions or sophisticated equipment, make it a practical field tool. In fact, the World Health Organization (WHO) has recently proposed WBA plus cytokine/chemokine quantification as a possible replacement for LST [20]. However, no epidemiological studies involving the use of such tests to estimate the prevalence of asymptomatic *Leishmania* infection have been published.

The largest outbreak of leishmaniasis in Europe to date, occurred in 2009 in the south-west of the Madrid region, Spain [21]. Most people affected lived in the town of Fuenlabrada, particularly in the north of the town, near the Bosque Sur park. This park, with its many hares and rabbits, supports a high-density population of sand flies [22]. Arce et al. reported 446 cases of cutaneous and visceral leishmaniasis between 2009 and 2012 [21], a figure that increased to a total of 758 cases in January 2018 [22,23]. Sex, distance from residence to the Bosque Sur park, immunosuppression and conversion from agricultural land to urban parkland (leading to presence of rural elements in the environment) were all associated with the clinical form of the disease [21,24]. However, the risk factors associated with the active form are not necessarily the same as those associated with asymptomatic or subclinical infection, and they need to be known for a better understanding of the dynamics of *Leishmania* transmission.

The aim of the present study was to determine the prevalence of, and risk factors for, asymptomatic *Leishmania* infection in Fuenlabrada following the 2009 outbreak, in order to inform future targeted public health measures.

## Methods

### Study design

A cross-sectional study was conducted from January to July 2015 within the framework of the Madrid region's V Seroepidemiological Survey (one of the information systems within the region's Epidemiological Surveillance Network). The target population was people living in Fuenlabrada. The study sample was formed of people attending public primary healthcare centres, understood to represent 'basic health zones' (BHZ) (as denominated by the Region of Madrid), in the field work period. A total of 804 people were recruited from among all the BHZs in Fuenlabrada (A: El Naranjo; B: Castilla la Nueva; C: Cuzco; D: Alicante; E: Panaderas; F: Francia; G: Parque Loranca), by random sampling of the following age groups: < 2, 2–5, 6–10, 11–15, 16–20, 21–30, 31-40, 41–60 and > 60 years. The annual mean incidence of disease (32.7 cases/100,000 population) was adopted as the threshold between two strata, grouping four high-incidence BHZs (A, B C and D) into stratum 1, and three low-incidence BHZs (E, F and G) into stratum 2. All persons with a condition that might cause immunosuppression, or who had experienced any clinical episode of leishmaniasis, were excluded.

A sample of peripheral blood was collected from each participant, all of whom completed a questionnaire (answers were collected by trained interviewers), recording their age, sex, country of birth, income level, medical history and their knowledge of health problems (i.e. immunosuppressant intake, infections such as HIV or hepatitis) and preventive measures against leishmaniasis. Basic data were collected from people who declined to participate, in order to control for possible sample bias.

### Detection of infection

#### Blood collection, serology and immunofluorescent antibody titres

Whole blood samples were collected in heparinised tubes for *Leishmania* DNA isolation and PCR, and for WBA with IL-2 quantification in SLA-stimulated plasma. Serum from blood samples (stored in EDTA-containing tubes) was used for the detection of anti-*Leishmania* antibodies.

DAT was performed using direct agglutination test for serodiagnosis of visceral leishmaniasis (ITMA-DAT/VL) (Prince Leopold Institute of Tropical Medicine, Antwerp, Belgium) following the manufacturer’s recommendations. Serum samples with a titre of 1:1,600 were considered positive.

IFAT were determined using 1 µl plasma samples following a standard method [25]. The antigen was prepared from *L. infantum* promastigotes (reference strain MHOM/FR/78/LEM-75), and antibody binding detected using fluorescein isothiocyanate-conjugated sheep anti-human IgG (heavy and light chains). The threshold titre for positivity was ≥ 1/80.

#### DNA extraction and kinetoplast PCR

*Leishmania* DNA was extracted from 100 μl of guanidine-treated peripheral blood by conventional phenol-chloroform extraction, and eluted in 100 μl sterile distilled water, as previously described [26]. For the PCR reaction, the primer pair M1/M2 targeting a partial kinetoplast DNA (kDNA) minicircle sequence was used.

#### Preparation of soluble *Leishmania infantum* antigen for whole blood stimulation assays

*L. infantum* antigen extract was prepared from stationary phase promastigote cultures (JPC strain, MCAN/ES/98/LLM-722) as previously described [27]. Briefly, parasites resuspended in lysis buffer (50 mM Tris/5 mM EDTA/HCl, pH 7) were subjected to three rapid freeze/thaw cycles followed by three 40 W sonicator pulses (20 s each). Two consecutive centrifugations at 27,000 x *g* for 20 min at 4 °C were then performed, and the supernatant collected, aliquoted, and stored at −80 °C until use. Protein quantification was performed using the Bradford method employing the Bio-Rad Protein Assay Kit (Bio-Rad, Hercules, CA, United States (US)).

#### Whole blood stimulation assay

Whole blood samples were stimulated as previously described [15]. Briefly, for each sample, an aliquot of blood (500 μL) was placed on its own in a tube (negative control), and another in a tube containing 10 μg/mL SLA; both were then incubated at 37 °C for 24 h. After centrifugation at 2,000 x *g* for 10 min, the supernatants were collected and stored at −20 °C for cytokine analysis. The IL-2 cut-off concentration was 50.37pg/mL [15].

#### Cytometric quantification of IL-2

IL-2 was determined in 50 μl of plasma from SLA-stimulated whole blood using the BD Cytometric Bead Array Human Flex Set (Becton Dickinson Biosciences, San Jose, CA, US) following the manufacturer’s instructions. Briefly, 50 μl of the plasma from the SLA-stimulated whole blood of each individual was incubated for 1 hour at room temperature with 50 μl of capture beads. After incubation, 50 μl of the detection antibody was added and the mixture allowed to react for 2 h at room temperature. Data were acquired using a FACSCalibur flow cytometer and analysed using the Flow Cytometric Analysis Programme Array (Becton Dickinson Biosciences). Results were expressed (in pg/mL) as the difference between the SLA-stimulated and control plasma IL-2 concentrations.

### Data analysis

The prevalence of *Leishmania* asymptomatic infection was estimated for each BHZ, determined by a positive result for DAT, IFAT, PCR or WBA with IL-2 quantification in the absence of any sign or symptom of leishmaniasis. The population of each BHZ was obtained from the Madrid Region's Institute of Statistics. Appropriate weightings were applied to correct deviations of the population structure of each BHZ stratum.

To examine the relationship between asymptomatic infection and vicinity to the initial leishmaniasis outbreak area, the primary healthcare centres for each BHZ were plotted on a digital map using the ArcGIS v.10 geographical information system (ESRI, Redlands, CA, US).

The strength of the associations between the IL-2 concentration in SLA-stimulated plasma and the risk factors associated with infection were estimated by calculating the odds ratios (OR) plus 95% confidence intervals (CI), and the corresponding p values. Bivariate and multivariate logistic regression analyses were performed to identify the risk factors associated with *Leishmania* asymptomatic infection; the categorical variables contemplated were age, sex, place of residence (stratum), place of birth, contact with dogs, contact with sick dogs or cats, type of residence, use of insecticides at home or when out walking, income and education level. Those variables showing a significant association with prevalence (an OR with a p value of < 0.10) in bivariate analysis were included in the multivariate model.

### Ethical considerations

The study was approved by the clinical research ethics committee of the Hospital Ramon y Cajal, and the Central Research Commission for Primary Care Management (registration number 259/14). All participants provided their informed consent to be included in the study.

## Results

### Description of the population

Of the 804 participants who gave consent to be included in the study and for whom blood samples were collected, seven were excluded according to the criteria specified in the methods and 797 filled in the questionnaire; 95.6% of these (762/797) were born in Spain and 4.4% (35/797) in other countries; 92.5% (737/797) lived in apartments, and 7.5% (60/797) in houses. Only 38.3% (305/797) reported having dogs or cats as pets, 38.4% (306/797) never used insecticides at home, 56.1% (447/797) used them occasionally, and 5.5% (44/797) used them frequently. When out walking and/or exercising, 80.2% (639/797) did not use repellents to protect themselves from sand fly bites, 16.7% (133/797) used them occasionally, and 3.1% (25/797) used them frequently. Around 70% of those who filled in the part of the survey on the economic aspects (281/398) had a net monthly income of over EUR 1,051 and had completed secondary education.

### Prevalence of asymptomatic infection

None of the 804 participants returned positive PCR results for *Leishmania* in blood. The prevalence of asymptomatic infection as measured by IFAT was 0.1% and by DAT 1.1% (Table 1). However, 20.7% of the individuals tested showed high SLA-stimulated plasma IL-2 concentrations.

**Table 1:** Prevalence of asymptomatic *Leishmania* infection for the different BHZs by laboratory test performed, cross-sectional study, Fuenlabrada (Madrid), Spain, 2015 (n = 804)

Stratum (BHZ)	N	Molecular test			Serological test						Cellular test		
		PCR			IFAT			DAT			WBA		
		n	Prevalence (%)	95% CI	n	Prevalence (%)	95% CI	n	Prevalence (%)	95% CI	n	Prevalence (%)	95% CI
1 (A to D)	469	0	0	(0 to 0)	1	0.19	(-0.19 to 0.58)	5	1.26	(0.04 to 2.49)	124	26.87	(22.15 to 31.59)
2 (E to G)	335	0	0	(0 to 0)	0	0	(0 to 0)	3	0.84	(-0.26 to 1.94)	40	12.12	(8.19 to 16.04)
Total	804	0	0	(0 to 0)	1	0.11	(-0.11 to 0.34)	8	1.09	(0.24 to 1.93)	164	20.71	(17.45 to 23.97)

BHZ: basic health zone; CI: confidence interval; DAT: direct agglutination test; IFAT: immunofluorescent antibody titre; N: total number of individuals; n: number of individuals with a positive test; WBA: whole blood assay with IL-2 quantification.

Basic health zones: A: El Naranjo; B: Castilla la Nueva; C: Cuzco; D: Alicante; E: Panaderas; F: Francia; G: Parque Loranca.

Stratum 1 consists of BHZs A to D, located near the Bosque Sur park.

Stratum 2 consists of BHZs E to G, situated at ca 5.3 km from Bosque Sur park.

**Figure 1:**
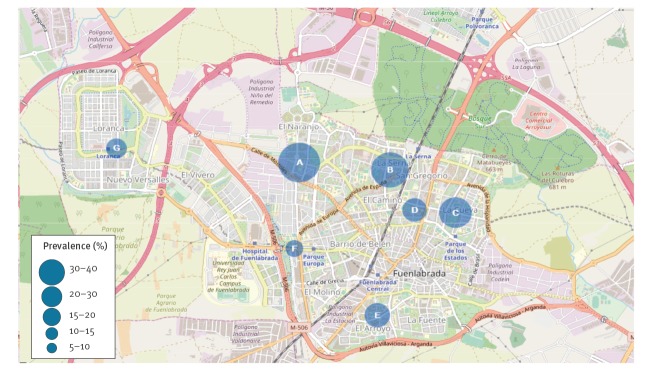
Spatial distribution and prevalence of asymptomatic individuals by basic health zones, cross-sectional leishmaniasis study, Fuenlabrada (Madrid), Spain, 2015

The following results are based on WBA with IL-2 quantification. The prevalence of asymptomatic infection was greatest among the population living in an area bordering the Bosque Sur park where the focus of infection was identified during the 2009 outbreak, and the lowest values were recorded in the main urban area (Figure 1).

Basic health zones: A: El Naranjo; B: Castilla la Nueva; C: Cuzco; D: Alicante; E: Panaderas; F: Francia; G: Parque Loranca.

**Figure 2:**
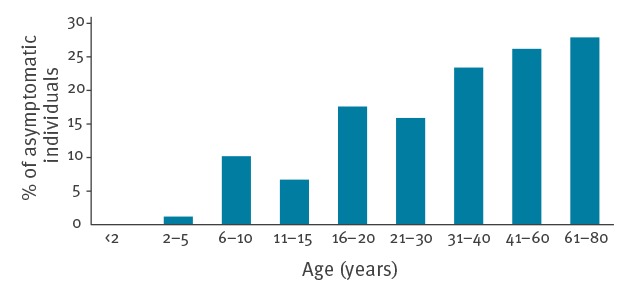
Distribution of asymptomatic *Leishmania* infections by age group, cross-sectional study, Fuenlabrada (Madrid), Spain, 2015 (n = 804)

Bivariate analysis showed that prevalence of asymptomatic infection increased remarkably with age; the prevalence for the > 60 years age group reached 28.6% (Figure 2 and Table 2). Differences were high between the 2–5-year age group and all other age groups but they were not statistically significant for the 6–10 and 11–15 year age groups. Greater prevalence was also seen among men compared to women, people living in a detached house, and people living close to the Bosque Sur park (stratum 1).

**Table 2:** Prevalence of asymptomatic *Leishmania* infections as determined by WBA with IL-2 quantification, by categorical variables, Fuenlabrada (Madrid), Spain, 2015 (n = 804)

Variable	n	%	Prevalence (%)	95% CI	cOR	95% CI	aOR	95% CI
**Age (years)**								
< 2	56	7.0	0	0 to 0	NA		NA	
2–5	66	8.2	1.2	-1.2 to 3.6	Ref		Ref	
6–10	48	6.0	10.2	1.7 to 18.8	9.3	1.0 to 83.3	9.6	1.0 to 90.7
11–15	52	6.5	6.7	0.2 to 13.2	5.9	0.6 to 55.1	6	0.6 to 57.4
16–20	51	6.3	17.6	6.5 to 28.8	17.6	2.1 to 147	16.6	1.9 to 143.4
21–30	129	16.0	17.3	10.5 to 24.2	17.2	2.2 to 131.9	16	2.0 to 127
31–40	123	15.3	23.4	15.7 to 31	25	3.3 to 189.8	28	3.6 to 220.1
41–60	145	18.0	26.4	19.2 to 33.6	29.4	3.9 to 221	32.1	4.2 to 247.7
61–80	134	16.7	28.6	20.9 to 36.4	32.9	4.4 to 247.3	37.9	4.9 to 293.4
**Sex**								
Female	410	51.0	13.2	9.5 to 16.9	Ref		Ref	
Male	394	49.0	28.2	23.1 to 33.4	2.6	1.7 to 3.9	2.9	1.9 to 4.6
**Stratum (BHZ)**								
2 (E–G)	335	41.7	12.2	8.3 to 16.2	Ref		Ref	
1 (A–D)	469	58.3	26.9	22.2 to 31.6	2.6	1.7 to 4.1	2.7	1.7 to 4.2
**Place of birth**								
Spain	762	95.6	21.5	18 to 24.9	Ref		Ref	
Other countries	35	4.4	7.2	-1.1 to 15.5	0.3	0.1 to 1	0.3	0.1 to 1.1
**Contact with dogs**								
No	492	61.7	18.3	14.3 to 22.3	Ref		Ref	
Yes	305	38.3	24.6	19 to 30.1	1.5	1.0 to 2.2	1.6	1.0 to 2.5
**Contact with sick dogs or cats**								
No	228	97.9	24.2	17.8 to 30.7	Ref		Ref	
Yes	5	2.1	44.3	-4.4 to 93	2.5	0.3 to 18.5	1.3	0.1 to 14
**Type of house**								
Apartment	737	92.5	19.5	16.1 to 22.8	Ref		Ref	
Detached house	60	7.5	34.5	21 to 48	2.2	1.2 to 4.1	2.9	1.4 to 5.7
**Use of insecticide at home**								
Never	306	38.4	24.1	18.6 to 29.7	Ref		Ref	
Occasionally	447	56.1	17.7	13.6 to 21.9	0.7	0.4 to 1.0	0.6	0.4 to 0.9
Always	44	5.5	28.6	13.4 to 43.9	1.3	0.6 to 2.8	1.0	0.4 to 2.4
**Use of insecticide while walking outside**								
Never	639	80.2	21.5	17.8 to 25.2	Ref		Ref	
Occasionally	133	16.7	16.6	9 to 24.3	0.7	0.4 to 1.3	1	0.6 to 1.9
Always	25	3.1	22.1	1.7 to 42.5	1.0	0.3 to 3.4	1.4	0.4 to 5
**Income level (EUR)**								
< 800	2	7.3	12.5	-1.6 to 26.6	Ref		Ref	
801–1,050	75	18.8	33.3	21 to 45.6	3.5	0.9 to 14.3	3.6	0.8 to 16.4
1,051–1,850	183	46.0	19.2	12.3 to 26	1.7	0.4 to 6.5	1.7	0.4 to 7.2
1,851–2,700	98	24.6	19.7	10.2 to 29.2	1.7	0.4 to 7.1	1.6	0.3 to 7.5
> 2,700	13	3.3	14.7	-4.7 to 34	1.2	0.2 to 9	1.3	0.1 to 12.7
**Level of education**								
Primary	372	46.6	18.4	NA	Ref		Ref	
Secondary	189	23.7	21.1	NA	1.2	0.7 to 2.0	0.9	0.5 to 1.6
Bachelor	155	19.4	25.8	NA	1.5	0.9 to 2.6	1.1	0.6 to 2.0
Higher degrees	82	10.3	15.0	NA	0.8	0.4 to 1.7	0.7	0.3 to 1.6

aOR: adjusted odds ratio; BHZ: basic health zone; CI: confidence interval; cOR: crude odds ratio; OR: odds ratio; NA: not applicable; Ref: reference group; WBA: whole blood assay.

Basic health zones: A: El Naranjo; B: Castilla la Nueva; C: Cuzco; D: Alicante; E: Panaderas; F: Francia; G: Parque Loranca.

Stratum 1 consists of BHZs A to D, located near the Bosque Sur park.

Stratum 2 consists of BHZs F to G, situated further away from Bosque Sur park.

All variables identified as statistically associated with prevalence in bivariate analysis were included in multivariate analysis, along with occasional use of insecticide at home (entered as a protective factor; OR _occasionally /never_ = 0.6 (95% CI: 0.4–0.9)).

## Discussion

Climate change and the lack of efficient leishmaniasis control measures have led to an increase in the number of cases of active disease recorded worldwide [20,28], with the appearance of unusual outbreaks. The detection of asymptomatic *Leishmania* infections in endemic areas is vital to know the true level of parasite transmission and the total number of infected individuals. In this epidemiological study, we report the prevalence of asymptomatic *Leishmania* infection and associated risk factors, in the post-outbreak area of Fuenlabrada (Madrid), Spain.

Traditional cell immunity-based LST analysis has in the past returned a mean prevalence value for asymptomatic *L. infantum* infection of 19.3% (31/161) for an active focus of VL in Tbilisi, Georgia, in 2014 [9], and of 23.1% (150/649) for an outbreak in Barbar El Fugara, Ethiopia, in 1995 [10]. LST is, however, currently prohibited for use in humans in many countries. The present study is the first to use WBA plus IL-2 quantification to determine the prevalence of asymptomatic infection. The value of 20.7% (143/804) returned for the study population of Fuenlabrada suggests this technique might be recommended for estimating the prevalence of asymptomatic individuals in field trials [20].

WBA plus IL-2 quantification also detected differences in the prevalence of asymptomatic infection between the BHZs within the Fuenlabrada area. The values were highest for the stratum 1 BHZs in the area bordering the park, where the original focus of infection was identified; values were lower for the stratum 2 BHZs which lay at greater distances. Similarly, in the above-mentioned Georgian study [9], the highest prevalence value (19.3%) was recorded in a district bordered by green parks, hills and forest. Urbanisation has affected the eco-epidemiological features of the infection and has had a significant impact on its distribution and re-emergence [24].

In an exhaustive work performed in the Department of Alpes-Maritimes in France, LST revealed a gradient of prevalence for asymptomatic infection ranging from 12% to 38% [11], matching the distribution of clinical cases. Similarly, the presented results match the distribution of active cases of VL detected during the 2009 outbreak [21]. The ability to use WBA plus IL-2 quantification as a cell immunity test for studying the spatial distribution of asymptomatic cases reinforces its value in epidemiological surveys. In fact, this non-invasive and non-sensitising simple blood stimulation assay was recently proposed as a replacement for LST by the WHO [20].

In the present study, the results for asymptomatic infection as measured serologically by IFAT and DAT were very low compared with those returned by WBA plus IL-2 quantification (1.1% vs 20.7%). Although western blot analysis of antibodies to *L. infantum* antigens is considered more sensitive than IFAT and DAT, it is not currently performed in large epidemiological studies because it is expensive, laborious and time-consuming [29]. To improve the sensitivity of detection of asymptomatic individuals, most studies in *Leishmania*-endemic areas use two or more indirect tests. Although two of the most widely used serological methods were included in the present study, the results did not match those obtained using WBA plus IL-2 quantification. This is associated with the fact that, whereas cell immunity is commonly retained for several years, perhaps even throughout an individual’s life [14], *Leishmania*-specific antibody levels tend to wane over time [30]. In fact, it has been demonstrated that cellular tests return higher *Leishmania* asymptomatic infection rates than serological or molecular analyses in epidemiological surveys [12,13]. The inclusion of cell immunity-based tests such as WBA plus IL-2 quantification would therefore be beneficial in field assays for asymptomatic *Leishmania* infection. Underestimates of the real prevalence might be obtained if only serological methods were used, influencing the control measures that might be adopted and the assessment of their impact.

Asymptomatic infection in immunocompetent individuals is generally accompanied by very low to undetectable parasitaemia [8]. In the present study, none of the 804 participants returned positive PCR results for *Leishmania* in blood. It might be wondered whether PCR would be more sensitive if buffy coat rather than whole blood samples were used (despite the difficulty of preparing buffy coat samples under field conditions). However, while some papers report different findings, a recent meta-analysis of 40 studies has shown no significant differences in diagnostic accuracy when using whole blood or buffy coat samples [24]. PCR is therefore not commonly used to identify asymptomatic individuals [31]. Indeed, the literature contains few studies that examine the prevalence of immunocompetent asymptomatic individuals via PCR. However, PCR has been used to determine the prevalence of asymptomatic infection by examining blood from blood banks, with values of 1.7% (33/2,000) reported from Greece [32], 5.9% (18/304) from Spain [33], 2.8% (16/565) from France [34], and 0.3% (4/1,449) from Italy [35]. In field studies undertaken in *L. infantum*-endemic areas, PCR methods estimated asymptomatic infection prevalence values of 10.0% (80/802) in Iran [36] and 7.9% (8/101) in Brazil [12]. The latter work also estimated the prevalence of asymptomatic infection by LST, which returned values of 71.3% (62/87) in this permanently high transmission area [12].

The xenodiagnosis of asymptomatic *Leishmania*-infected individuals has only been described in HIV-positive patients, but revealed the potential of this asymptomatic, immunodeficient population to act as a reservoir of *L. infantum* [37]. It might be wise, however, to look beyond HIV-positive patients to those with other immunocompromising conditions, such cancer, solid organ transplants, or treatment with immunosuppressant drugs for other reasons [38].

It is important to know the risk factors involved in *Leishmania* infection if control programmes are to be effective. In the present study, age, sex, distance to the Bosque Sur park and type of housing, all appeared to influence exposure to and infection with *Leishmania*. These results agree with those reported by Arce et al., who described most of these factors as related to the incidence of clinical cases in the same area [21]. In the present study, the prevalence of asymptomatic infection increased with age compared with the 2–5-years age group (reaching statistical significance for the age groups above 16 years), which suggests a non-exclusive role for the 2009 outbreak in determining the population with asymptomatic infection. In studies performed after the VL outbreak that occurred in northern Italy in 1971, all age groups showed a similarly high positivity rate by LST in the outbreak area, suggesting a simultaneous exposure to infection. However, in a Sicilian endemic area taken as a control, age-specific LST rates showed an increasing positive rate with age [39].

In most endemic foci of leishmaniasis, active cases of disease have been reported more commonly in men than in women [1,35,40]. In Fuenlabrada, from 2009 to 2012, the percentages of infected men and women with active disease were 69.5% and 30.5% respectively [21], matching the present sex distribution for asymptomatic infection. This difference might be explained by men more often undertaking activities that might expose them to the vector.

In Spain, dogs are the main reservoir of *L. infantum*, and in the present study 38.3% of participants reported having had contact with dogs in their domestic or peridomestic environment; 24.6% (of the 38.3%) developed a cellular immune response against *Leishmania*. Contact with dogs would thus appear to increase the likelihood of becoming an asymptomatic carrier. Some 7.6% of the exposed population lived in houses, and 34.5% of these had an asymptomatic infection. The presence of gardens, more open spaces and being surrounded by green areas increases the likelihood of asymptomatic infection. Arce et al. reported this to be an important risk factor for developing the disease [21].

The present results suggest that the occasional use of insecticides with repellent action in the domestic environment may prevent humans and dogs being bitten by sand flies, reducing the risk of in-home transmission. However, the frequent use of repellents was not associated with statistically significant protection. In this respect, collected data did not provide conclusive results which can be due to the use and type of repellent and/or time and type of exposure to the sand fly and its behaviour. *Phlebotomus perniciosus* is the only vector in the affected area, where it appears at high density (193.6 specimens/m^2^) [22]. Although it exhibits exophilic behaviour, *P. perniciosus* is strongly attracted by light [41], and it might enter homes. We hypothesize that it might also play some role in the transmission of this emergent form of infection in an urban environment, although no intra-domiciliary cycle was established in the present outbreak.

Environmental control measures were taken to control the outbreak in Fuenlabrada, such as improvements in sanitation, vector control measures in the main risk areas, and control of rabbit and hare numbers [21]. However, periodic surveys of *Leishmania* infection in the exposed population in this area, and in new areas where the transmission of *L. infantum* to humans is detected, are required if we are to know the infection rate.

The present study shows that a high percentage of the population in Fuenlabrada has been infected with the *Leishmania* parasite. Whereas most immunocompetent individuals will develop no disease, immunosuppression is a well-established risk factor for its appearance. In Europe, the use of immunomodulatory drugs to treat different conditions is increasing. However, no screening to detect the potentially *Leishmania*-infected but asymptomatic population is performed, and immunosuppression may reactivate a latent infection, ending in active VL.

In recent years, the incidence of imported leishmaniasis has increased in non-endemic European countries, a consequence of increased travel and immigration from endemic regions [42-44], with over 50% of those infections acquired in either Spain or Greece. Indeed, it is known that several Swedish patients undergoing tumor necrosis factor (TNF)-α modulating therapy became infected with leishmaniasis while visiting Spain [45]. Physicians outside of Europe's *Leishmania*-endemic area should consider imported leishmaniasis when a patient's symptoms and travel history suggest this, especially if the patient is already being treated with immunosuppressants.

In conclusion, WBA plus IL-2 quantification was found to be a useful tool in epidemiological surveys. This easy-to-use technology returned a high prevalence of asymptomatic infection in a post-outbreak area in Spain. In addition, it helped determine age, sex, distance to the focus of infection and type of home to be risk factors associated with asymptomatic infection. The true level of parasite transmission and the total number of infected individuals might be underestimated if only serological methods are used in epidemiological studies.
